# The genome sequence of the Garden Ectemnius, *Ectemnius cavifrons* (Thomson, 1870) (Hymenoptera: Crabronidae)

**DOI:** 10.12688/wellcomeopenres.27971.1

**Published:** 2026-09-28

**Authors:** Liam M. Crowley, University of Oxford and Wytham Woods Genome Acquisition Lab, Darwin Tree of Life Barcoding Collective, Wellcome Sanger Institute Tree of Life Management, Samples and Laboratory team, Wellcome Sanger Institute Scientific Operations: Sequencing Operations, Wellcome Sanger Institute Tree of Life Core Informatics team, Tree of Life Core Informatics collective, Darwin Tree of Life Consortium

**Affiliations:** 1 University of Oxford, Oxford, Oxfordshire, England, UK

**Keywords:** Ectemnius cavifrons, Garden Ectemnius, genome sequence, chromosomal, Hymenoptera

## Abstract

We present a genome assembly from an individual male *Ectemnius cavifrons* (Garden Ectemnius; Arthropoda; Insecta; Hymenoptera; Crabronidae). The genome sequence has a total length of 225.31 megabases. Most of the assembly (93.48%) is scaffolded into 14 chromosomal pseudomolecules. The mitochondrial genome has also been assembled, with a length of 21.69 kilobases. Gene annotation of the haploid assembly by Ensembl identified 10,644 protein-coding genes. This assembly was generated as part of the Darwin Tree of Life project, which produces genomes for eukaryotic species found in Britain and Ireland.

## Species taxonomy

Eukaryota; Opisthokonta; Metazoa; Eumetazoa; Bilateria; Protostomia; Ecdysozoa; Panarthropoda; Arthropoda; Mandibulata; Pancrustacea; Altocrustacea; Allotriocarida; Hexapoda; Insecta; Dicondylia; Pterygota; Neoptera; Eumetabola; Endopterygota; Hymenoptera; Apocrita; Aculeata; Apoidea; Crabronidae; Crabroninae; Crabronini; Crabronina; *Ectemnius*; *Ectemnius cavifrons* (Thomson, 1870) (NCBI:txid1518028)

## Background

Digger wasps (Crabronidae) are solitary wasps that nest in the ground, hollow stems or dead wood ( Brock, 2021). The genus *Ectemnius* occurs worldwide; its members nest in rotten wood or plant stems and take flies as prey. Adults are often seen visiting umbellifer flowers ( Richards, 1980).

*Ectemnius cavifrons* (Thomson, 1870), the garden ectemnius, is one of the larger British members of the genus, measuring 11–16 mm in length. It is black with yellow markings, including bands and spots on the abdomen ( Brock, 2021). Males have toothed antennae, with a deep notch beneath two teeth on the third segment, and golden hairs on the centre of the clypeus ( Brock, 2021; Richards, 1980).

The species occurs in Europe and northern Africa, with its range extending across Asia to Korea, Japan and south-eastern Asia ( Ruchin & Antropov, 2019). In Britain, it is common and occurs in woodland, hedgerows, parks and gardens. Nests are made in dead wood and provisioned chiefly with hoverflies (Syrphidae) ( Brock, 2021; Richards, 1980).

We present a chromosome-level genome sequence for *Ectemnius cavifrons*, produced using the Tree of Life pipeline. The sequence is based on a specimen collected from Wytham Woods, Oxfordshire, UK (Figure 1). This assembly was generated as part of the Darwin Tree of Life Project, which aims to generate high-quality reference genomes for all named eukaryotic species in Britain and Ireland to support research, conservation, and the sustainable use of biodiversity ( Darwin Tree of Life Project Consortium, 2022).

Although numerous genomes have been sequenced for the family Crabronidae, this assembly provides the first chromosome-level genome assembly for *Ectemnius cavifrons*, enabling comparative analyses (data obtained via NCBI datasets, O’Leary *et al.*, 2024). This is currently the only genome assembly available for this species. This assembly is the RefSeq reference assembly for this species.

**Figure 1.**
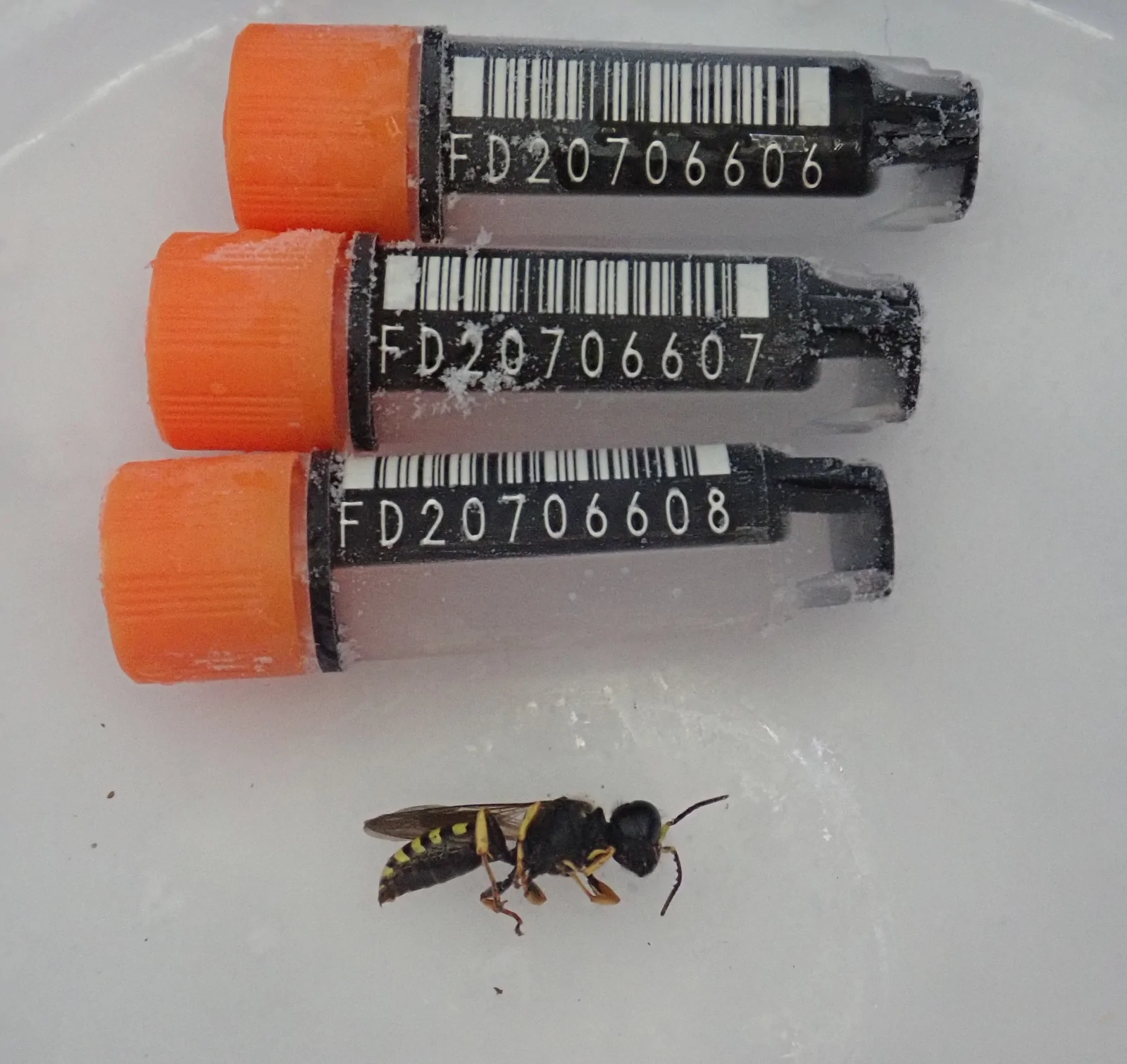
The *E. cavifrons* specimen (Ox001582, iyEctCavi1) used for genome sequencing.

## Methods

### Sample acquisition and DNA barcoding

Two adult *Ectemnius cavifrons* specimens were used: the male genome-sequencing specimen (specimen ID Ox001582, ToLID iyEctCavi1; Figure 1) and the female specimen used for Hi-C and RNA (specimen ID Ox001637, ToLID iyEctCavi2). Both were collected from Wytham Woods, Oxfordshire, UK (latitude 51.769, longitude −1.328) by Liam Crowley, who also identified them. The male was collected on 2021-07-14 and the female on 2021-07-08.

The initial identification was verified by an additional DNA barcoding process according to the framework developed by Twyford *et al.*. (2024). A small sample was dissected from the specimen and stored in ethanol, while the remaining parts were shipped on dry ice to the Wellcome Sanger Institute (WSI) (see the protocol). The tissue was lysed, the COI marker region was amplified by PCR, and amplicons were sequenced and compared to the BOLD database, confirming the species identification ( Crowley *et al.* , 2023). Following whole-genome sequencing, the relevant DNA barcode region was also used alongside the initial barcoding data for sample tracking at the WSI ( Twyford *et al.* , 2024). The standard operating procedures for Darwin Tree of Life barcoding are available on protocols.io.

### Nucleic acid extraction

High molecular weight (HMW) DNA extraction protocols developed by the Tree of Life Core Laboratory at the Wellcome Sanger Institute (WSI) are available on protocols.io ( Howard *et al.*, 2025). Tissue from the iyEctCavi1 sample was weighed and triaged to select the appropriate extraction workflow. For library DTOL13161575, thorax tissue was homogenised by PowerMash using a PowerMasher II tissue disruptor. HMW DNA was extracted using the Automated MagAttract v2 protocol. We used centrifuge-mediated fragmentation to produce DNA fragments in the 8–10 kb range, following the Covaris g-TUBE protocol for ultra-low input (ULI). Sheared DNA was purified by automated SPRI (solid-phase reversible immobilisation).

The concentration of the sheared and purified DNA was assessed with a NanoDrop spectrophotometer and a Qubit Fluorometer using the Qubit dsDNA High Sensitivity Assay kit. Fragment size distribution was evaluated by running the sample on the Femto Pulse system. For this sample, final post-shearing DNA measurements included a Qubit concentration of 2.64 ng/μL, an average fragment size of 9.7 kb and a yield of 2,051.40 ng. The 260/280 spectrophotometric ratio was 4.32, and the 260/230 ratio was 7.42.

RNA was extracted from abdomen tissue of the iyEctCavi2 specimen in the Tree of Life Laboratory at the WSI using the RNA Extraction: Automated MagMax™ *mir*Vana protocol. The RNA concentration was assessed with a NanoDrop spectrophotometer and a Qubit Fluorometer using the Qubit RNA Broad-Range Assay kit. RNA integrity was assessed using the Agilent RNA 6000 Pico Kit and Eukaryotic Total RNA assay.

### PacBio HiFi library preparation and sequencing

Library preparation and sequencing took place in the WSI Scientific Operations core. Library DTOL13161575 was prepared with the ULI PacBio HiFi workflow. Prior to library preparation, DNA was fragmented to approximately 10 kb. The library was prepared with the PacBio SMRTbell® Express Template Prep Kit 2.0 and gDNA Sample Amplification Kit. The sample was normalised to 20 ng DNA. Single-strand overhang removal, DNA damage repair and end-repair/A-tailing were followed by adapter ligation and a 0.85× pre-PCR clean-up with Promega ProNex beads.

The DNA was divided evenly between two PCRs, and each underwent a 0.85× post-PCR clean-up with ProNex beads. DNA concentration was measured with a Qubit Fluorometer v4.0 (Thermo Fisher Scientific) with the Qubit High Sensitivity Assay Kit, and fragment size was assessed using an Agilent Femto Pulse Automated Pulsed Field CE Instrument (Agilent Technologies) with the gDNA 55 kb BAC analysis kit. The two PCRs were pooled to provide at least 500 ng DNA in 47.4 μL. Following DNA damage repair, end-repair/A-tailing and hairpin-adapter ligation, the pooled sample underwent a 1× ProNex bead clean-up. Size selection was performed using a Sage Science PippinHT system, with the target fragment size determined by Femto Pulse analysis. Size-selected libraries were cleaned with 1.0× ProNex beads and normalised to 2 nM before sequencing.

The library was sequenced using the Sequel IIe system (Pacific Biosciences, California, USA). Library loading concentrations were in the range 40–135 pM. SMRT Link software (Pacific Biosciences) was used to configure and monitor the run and to perform primary and secondary data analysis.

### Hi-C

***Sample preparation and crosslinking***

Hi-C data were generated from the iyEctCavi2 head sample using the Arima-HiC v2 kit (Arima Genomics). Following the manufacturer’s instructions, tissue was fixed and DNA crosslinked using TC buffer to a final formaldehyde concentration of 2%. The tissue was homogenised using the Diagnocine Power Masher-II. Crosslinked DNA was digested with a restriction enzyme master mix, biotinylated, and ligated. Clean-up was performed with SPRISelect beads before library preparation. DNA concentration was measured with the Qubit Fluorometer (Thermo Fisher Scientific) and Qubit HS Assay Kit. The biotinylation percentage was estimated using the Arima-HiC v2 QC beads.

***Hi-C library preparation and sequencing***

Biotinylated DNA constructs were fragmented using a Covaris E220 sonicator and size-selected to 400–600 bp using SPRISelect beads. DNA was enriched with Arima-HiC v2 kit Enrichment beads. End repair, A-tailing, and adapter ligation were carried out with the NEBNext Ultra II DNA Library Prep Kit (New England Biolabs), following a modified protocol where library preparation occurs while DNA remains bound to the Enrichment beads. Libraries were amplified with KAPA HiFi HotStart mix and a custom Unique Dual Index (UDI) barcode set (Integrated DNA Technologies). Depending on sample concentration and biotinylation percentage determined at the crosslinking stage, libraries were amplified with 10–16 PCR cycles. Post-PCR clean-up was performed with SPRISelect beads. Libraries were quantified using the AccuClear Ultra High Sensitivity dsDNA Standards Assay Kit (Biotium) and a FLUOstar Omega plate reader (BMG Labtech).

Prior to sequencing, libraries were normalised to 10 ng/μL. Normalised libraries were quantified again to create equimolar and/or weighted 2.8 nM pools. Pool concentrations were checked using the Agilent 4200 TapeStation (Agilent) with High Sensitivity D500 reagents before sequencing. Libraries were sequenced on the Illumina NovaSeq 6000, generating paired-end 150-bp reads.

### RNA library preparation and sequencing

Libraries were prepared with the NEBNext® Ultra II Directional RNA Library Prep Kit for Illumina (New England Biolabs), following the manufacturer’s instructions. Poly(A) mRNA was isolated from 100 ng of total RNA using oligo(dT) beads, converted to cDNA, and uniquely indexed; 14 PCR cycles were performed. Libraries were prepared with an intended average fragment size of 100–300 bp. Libraries were quantified, normalised, and pooled equimolarly to a final concentration of 2.8 nM before dilution to 150 pM for loading. Sequencing was carried out on the Illumina NovaSeq 6000, generating paired-end reads.

### Genome assembly

Prior to assembly of the PacBio HiFi reads, a database of *k*-mer counts ( *k* = 31) was generated from the filtered reads using FastK. The *k*-mer frequency distribution was analysed with GenomeScope (version 2.1.0) ( Ranallo-Benavidez *et al.*, 2020), providing fitted point estimates of haploid genome size, heterozygosity and repeat content.

The HiFi reads were assembled using Hifiasm ( Cheng *et al.*, 2021) with the --primary and -l0 options. Internal Hifiasm purging was switched off with -l0. Haplotypic duplications were identified and removed using purge_dups ( Guan *et al.*, 2020). The Hi-C reads ( Rao *et al.* , 2014) were mapped to contigs using bwa-mem2 ( Vasimuddin *et al.*, 2019), and the contigs were scaffolded in YaHS ( Zhou *et al.*, 2023) with the --break option for handling potential misassemblies. The scaffolded assemblies were evaluated using Gfastats ( Formenti *et al.* , 2022), BUSCO ( Manni *et al.*, 2021) and MerquryFK ( Rhie *et al.*, 2020).

The mitochondrial genome was assembled using MitoHiFi ( Uliano-Silva *et al.* , 2023).

### Assembly curation

The assembly was decontaminated using the Assembly Screen for Cobionts and Contaminants ( ASCC) pipeline. The flat files and maps for curation were generated with TreeVal. Manual curation was conducted primarily in PretextView and HiGlass ( Kerpedjiev *et al.* , 2018). Scaffolds were visually inspected and corrected as described by Howe *et al*. (2021). Manual corrections included 39 breaks and 102 joins. This reduced the scaffold count by 25.3%, increased the scaffold N50 by 18.2%, and reduced the total assembly length by 1.6%. The curation process is described at sanger-tol/curation-resources. A Hi-C contact map of the haploid assembly was generated with PretextSnapshot.

### Assembly quality assessment

The MerquryFK tool ( Rhie *et al.*, 2020) was run in a Singularity container ( Kurtzer *et al.*, 2017) to evaluate *k*-mer completeness and assembly quality for the haploid assembly using the *k*-mer database ( *k* = 31) computed prior to genome assembly. The analysis outputs included assembly QV scores and completeness statistics.

The haploid assembly was assessed using BlobToolKit ( Challis *et al.*, 2020). PacBio reads were mapped to the assembly to derive sequence coverage, and BUSCO ( Manni *et al.*, 2021) was used to assess gene-set completeness. DIAMOND BLASTp searches of extracted BUSCO proteins and DIAMOND BLASTx searches of assembly sequences against UniProt Reference Proteomes ( Buchfink *et al.*, 2021), followed by BLASTn searches of sequences without DIAMOND BLASTx hits against the NCBI nt database ( Altschul *et al.*, 1990), supported taxonomic assignment. Coverage, sequence composition, taxonomic assignment and BUSCO results were integrated into a BlobDir for interactive inspection and visualisation. The snail and blob plots were rendered locally from this hosted BlobDir using BlobTk v0.8.3. The scaffold-length radial scale function for the snail plot was set to linear ( Challis & Blaxter, 2026). The blob plot used circular markers with area proportional to scaffold length ( Challis *et al.*, 2020).

## Genome sequence report

### Sequence data

PacBio sequencing of the *E. cavifrons* specimen generated 16.85 Gb (gigabases) from 1.55 million reads. These reads were used to assemble the genome. GenomeScope analysis using a *p* = 2 model gave a *k*-mer-based haploid genome size estimate of 199.65 Mb, with heterozygosity of 0.25% and repeat content of 14.80%; the full-model fit was 87.36% (Figure 2). The GenomeScope estimates guided expectations for the assembly. The NCBI assembly record reports 77× genome coverage. Hi-C sequencing produced 98.65 Gb from 326.65 million reads, which were used to scaffold the haploid assembly. RNA sequencing data were also generated and are available in public sequence repositories. Table 1 summarises the specimen and sequencing details.

**Figure 2.**
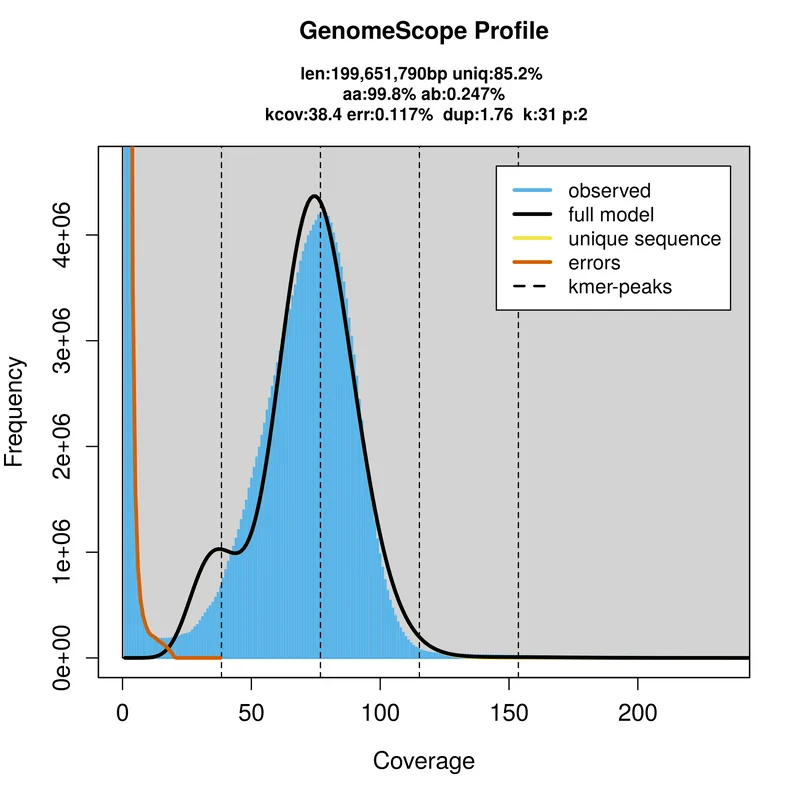
GenomeScope model fitted to the *k*-mer frequency distribution. The plot shows the observed and modelled *k*-mer spectra used to estimate haploid genome size, heterozygosity and repeat content from unassembled sequencing reads.

**Table 1.** Specimen and sequencing data for *E. cavifrons* (BioProject PRJEB66041)

**Platform**	**PacBio HiFi**	**Hi-C**	**RNA-seq**
**ToLID**	iyEctCavi1	iyEctCavi2	iyEctCavi2
**Specimen ID**	Ox001582	Ox001637	Ox001637
**BioSample (source individual)**	SAMEA10978752	SAMEA10978906	SAMEA10978906
**BioSample (tissue)**	SAMEA10978842	SAMEA10979235	SAMEA10979237
**Tissue**	thorax	head	abdomen
**Instrument**	Sequel IIe	Illumina NovaSeq 6000	Illumina NovaSeq 6000
**Library ID & run accession(s)**	DTOL13161575: ERR12055576	ERR12071250	ERR12071251
**Read count total**	1.55 million reads	326.65 million read pairs	26.20 million read pairs
**Base count total**	16.85 Gb	98.65 Gb	7.91 Gb

### Assembly statistics

The haploid genome was assembled as a single assembly. It has a total length of 225.31 Mb in 271 scaffolds (excluding organelle sequences), with 305 gaps, and its scaffold N50 is 14.99 Mb (Table 2). The scaffold auN for the haploid assembly is 15.1 Mb.

**Table 2.** Genome assembly data for *E. cavifrons*

**Genome assembly**	**Haploid assembly**
**Assembly name**	iyEctCavi1.1
**Assembly accession**	GCA_964023945.1
**Assembly level**	chromosome
**Span (Mb)**	225.31
**Number of chromosomes**	14
**Number of contigs**	576
**Contig N50 (Mb)**	1.3
**Number of scaffolds (excluding organelle sequences)**	271
**Scaffold N50 (Mb)**	14.99
**Scaffold auN (Mb)**	15.1
**Organelles**	Mitochondrial genome: 21.69 kb

Most of the assembly sequence (93.48%) was assigned to 14 chromosome-level scaffolds. These chromosome-level scaffolds, confirmed by Hi-C data, are named according to size (Figure 3; Table 3).

The mitochondrial genome was also assembled (length 21.69 kb, OZ030389.1). This sequence is included as a contig in the multifasta file of the genome submission and as a standalone record.

**Figure 3.**
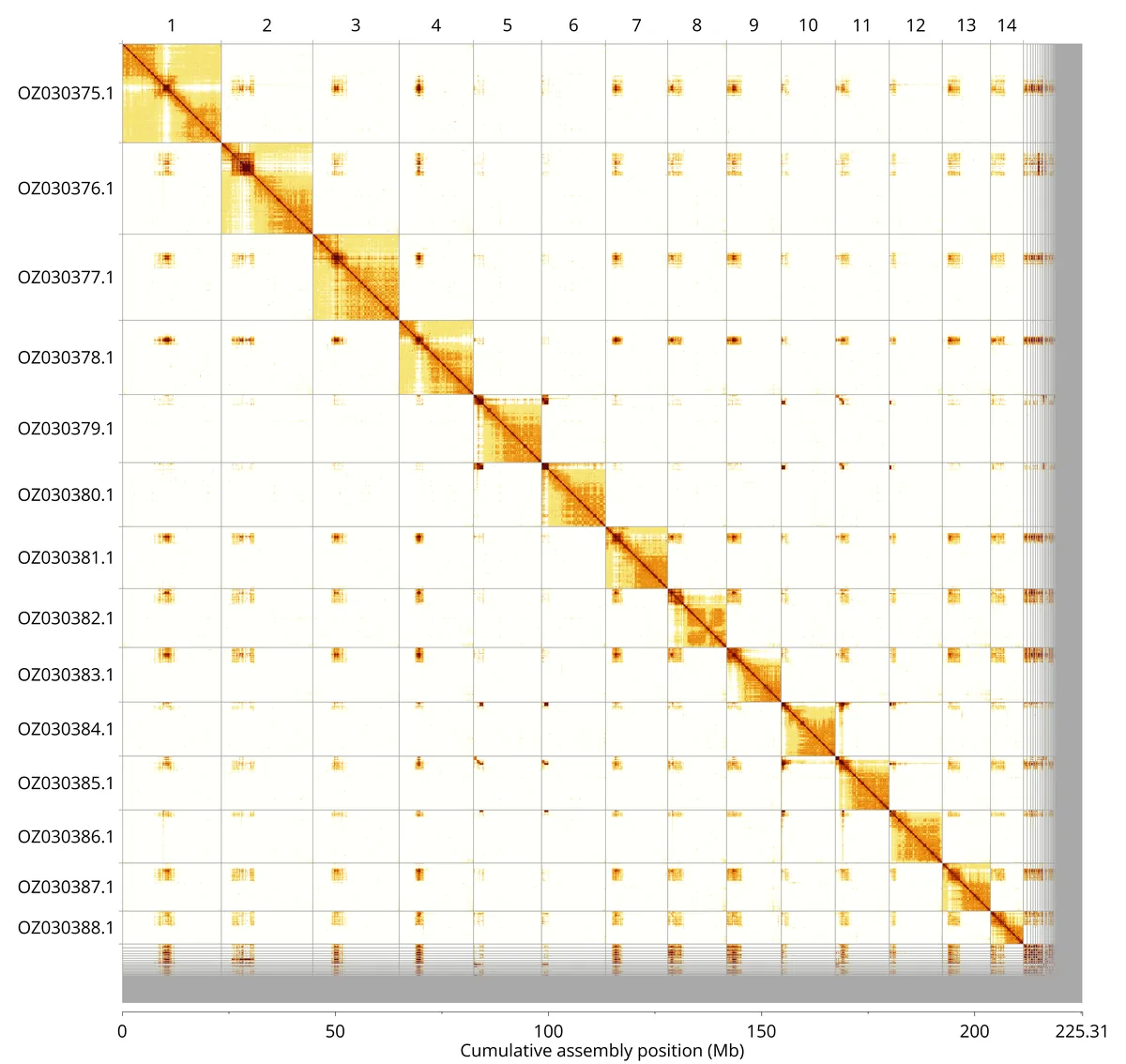
Hi-C contact map of the *E. cavifrons* haploid assembly. Assembled chromosomes are shown in order of size and labelled along the axes, with a megabase scale shown below. The plot was generated using PretextSnapshot.

**Table 3.** Chromosomal pseudomolecules in the haploid genome assembly of *E. cavifrons* iyEctCavi1

**INSDC accession**	**Molecule**	**Length (Mb)**	**GC%**
OZ030375.1	1	23.16	43
OZ030376.1	2	21.40	43.50
OZ030377.1	3	20.15	43.50
OZ030378.1	4	17.40	43
OZ030379.1	5	15.87	44
OZ030380.1	6	14.99	43
OZ030381.1	7	14.52	44
OZ030382.1	8	13.78	44.50
OZ030383.1	9	12.76	44.50
OZ030384.1	10	12.68	43
OZ030385.1	11	12.56	44.50
OZ030386.1	12	12.44	44
OZ030387.1	13	11.24	43.50
OZ030388.1	14	7.68	43.50

### Assembly quality metrics

The haploid assembly achieves an estimated QV of 63.6, and the *k*-mer completeness is 99.43% (Figure 4).

**Figure 4.**
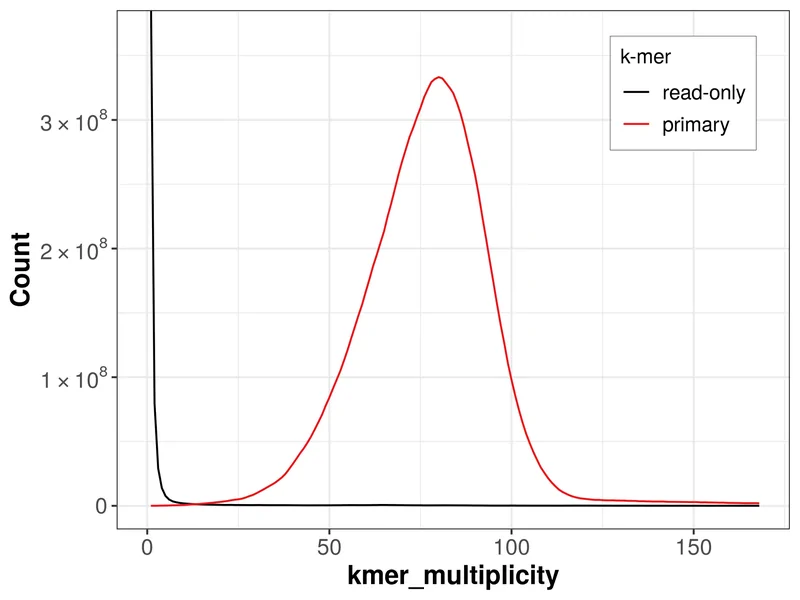
Evaluation of *k*-mer completeness using MerquryFK. This plot illustrates the recovery of *k*‐mers from the original read data in the final haploid assembly. The horizontal axis represents *k*‐mer multiplicity, and the vertical axis shows the number of *k*‐mers. The black curve represents *k*‐mers that appear in the reads but are not assembled, while the remaining curves correspond to *k*‐mers recovered in the haploid assembly.

BUSCO v.5.5.0 analysis of the haploid assembly using the hymenoptera_odb10 reference set ( *n* = 5,991) identified 95.7% of the expected gene set (single = 95.7%, duplicated = 0.0%).

The snail plot in Figure 5 provides a summary of assembly contiguity, cumulative scaffold count, sequence composition and BUSCO completeness for the haploid assembly ( Challis & Blaxter, 2026). The relative sizes of the longest scaffold, scaffold N50 and N90, together with the dark-grey scaffold-length distribution, show how much of the assembly is contained in long scaffolds. The outer composition track shows variation in GC, AT and N content, while the central track shows the cumulative scaffold count and the BUSCO inset summarises gene-set completeness.

The blob plot in Figure 6 displays scaffolds from the haploid assembly by GC proportion and base coverage. Marker area is proportional to scaffold length, and colour indicates taxonomic assignment ( Challis *et al.*, 2020).

**Figure 5.**
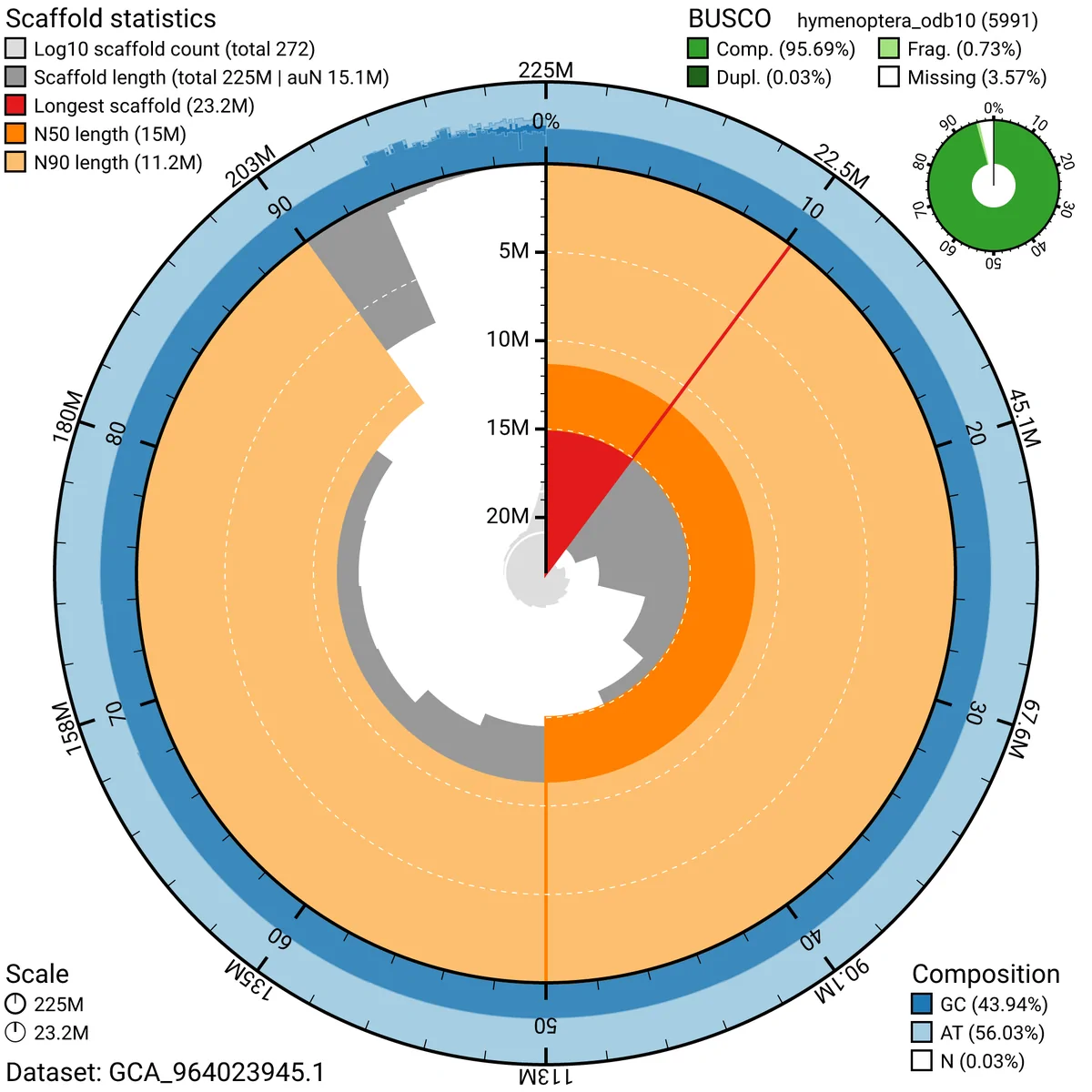
Assembly metrics for iyEctCavi1.1, the haploid assembly. The BlobToolKit snail plot provides an overview of assembly metrics and BUSCO gene completeness. The circumference represents the length of the haploid assembly, and the main plot is divided into 1,000 bins around the circumference. The outermost blue tracks display the distribution of GC, AT, and N percentages across the bins. Scaffolds are arranged clockwise from longest to shortest and are depicted in dark grey. Scaffold length is shown on a linear radial scale, scaled to the longest scaffold. Red shading indicates the longest scaffold, and the deeper orange and pale orange shading represent the N50 and N90 lengths. The summary statistics include scaffold auN, the area under the scaffold Nx curve. The light grey spiral at the centre shows the cumulative scaffold count on a logarithmic scale. A summary of complete, fragmented, duplicated, and missing BUSCO genes in the hymenoptera_odb10 set is presented at the top right. The assembly can be explored interactively in the BlobToolKit viewer.

**Figure 6.**
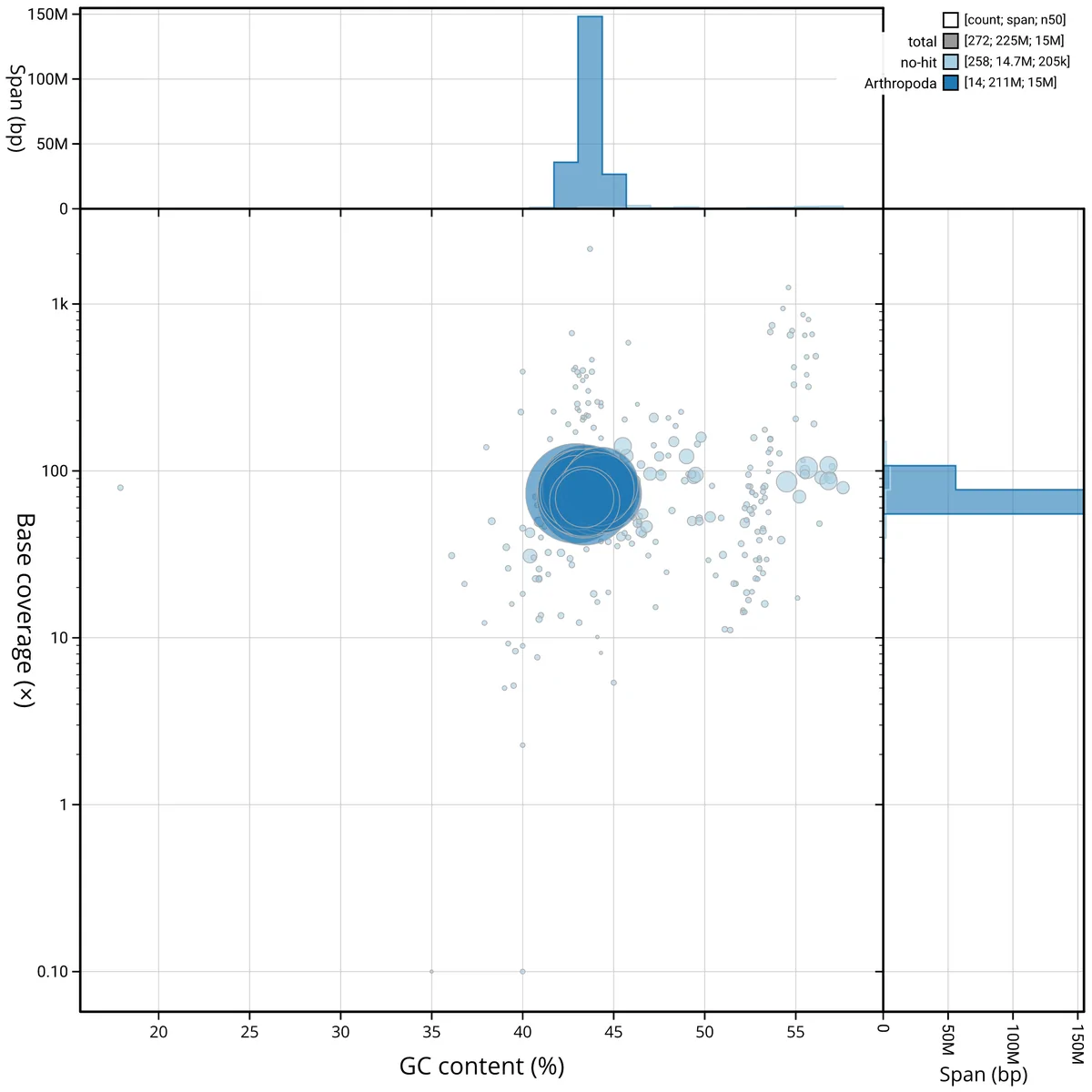
BlobToolKit blob plot for iyEctCavi1.1. The plot shows base coverage (vertical axis) and GC content (horizontal axis). Circles represent scaffolds; marker area is proportional to scaffold length, and colour indicates taxonomic assignment. The histograms along the axes display the total length of sequences distributed across different levels of coverage and GC content. The assembly can be explored interactively in the BlobToolKit viewer.

Table 4 lists the assembly metric benchmarks adapted from Rhie *et al.*. (2021) and the Earth BioGenome Project (EBP) Report on Assembly Standards. The three-part EBP metric, calculated for the haploid assembly, is **6.C.Q63**.

**Table 4.** Earth BioGenome Project summary metrics for the *E. cavifrons* assembly

**Measure**	**Value**	***Benchmark***
EBP summary (haploid assembly)	6.C.Q63	*6.C.Q40*
Contig N50 length	1.30 Mb	*> 1 Mb*
Scaffold N50 length	14.99 Mb	*= chromosome N50*
Consensus quality (QV)	Haploid assembly: 63.6	*≥ 40*
*k*-mer completeness	Haploid assembly: 99.43%	*> 90%*
BUSCO	C:95.7% [S:95.7%, D:0.0%], F:0.7%, M:3.6%, n:5,991	*S > 90%; D < 5%*
Percentage of assembly assigned to chromosomes	93.48%	*≥ 90%*

**Notes:** The EBP summary uses log10(Contig N50); chromosome-level (C) or log10(Scaffold N50); Q (Merqury QV). BUSCO v5.5.0 using the hymenoptera_odb10 lineage dataset: C=complete; S=single-copy; D=duplicated; F=fragmented; M=missing; n=orthologues.

### Genome annotation report

The *E. cavifrons* genome assembly (GCA_964023945.1) was annotated by Ensembl at the European Bioinformatics Institute (EBI). The annotation comprises 16,737 transcripts from 10,644 protein-coding genes and 450 non-coding genes. The average transcript length is 9,291.91 bp, with an average of 1.51 coding transcripts per gene and 6.89 exons per transcript. Further details of this annotation are available from the Ensembl annotation page.

### Data availability

European Nucleotide Archive: Ectemnius cavifrons. Accession number PRJEB66041; https://identifiers.org/ena.embl/PRJEB66041. The genome sequence is released openly for reuse. The *E. cavifrons* genome sequencing initiative is part of the Darwin Tree of Life Project ( PRJEB40665) and the Sanger Institute Tree of Life Programme ( PRJEB43745). All raw sequence data and the haploid assembly have been deposited in INSDC databases. Raw data and assembly accession identifiers are reported in Table 1 and Table 2.

Production code used in genome assembly at the WSI Tree of Life is available at https://github.com/sanger-tol. Table 5 lists software versions used in this study.

**Table 5.** Software versions and sources

**Software**	**Version**	**Source**
BLAST	2.14.0	https://ftp.ncbi.nlm.nih.gov/blast/executables/blast+/
BlobToolKit	4.3.9	https://github.com/genomehubs/blobtoolkit
BlobTk (hosted BlobDir)	0.5.1	https://github.com/genomehubs/blobtk
BlobTk (snail and blob plot renderer)	0.8.3	https://github.com/genomehubs/blobtk
BUSCO	5.5.0; 6.1.0	https://gitlab.com/ezlab/busco
bwa-mem2	2.2.1	https://github.com/bwa-mem2/bwa-mem2
DIAMOND	2.1.8	https://github.com/bbuchfink/diamond
fasta_windows	0.2.4	https://github.com/tolkit/fasta_windows
FastK	1.2	https://github.com/thegenemyers/FASTK
GenomeScope	2.1.0	https://github.com/tbenavi1/genomescope2.0
Gfastats	1.3.6	https://github.com/vgl-hub/gfastats
Hifiasm	0.16.1	https://github.com/chhylp123/hifiasm
HiGlass	1.13.4	https://github.com/higlass/higlass
MerquryFK	1.1.0-c1	https://github.com/thegenemyers/MERQURY.FK
Minimap2	2.24-r1122	https://github.com/lh3/minimap2
MitoHiFi	3.1	https://github.com/marcelauliano/MitoHiFi
MultiQC	1.14; 1.17 and 1.18	https://github.com/MultiQC/MultiQC
Nextflow	23.10.0	https://github.com/nextflow-io/nextflow
PretextSnapshot	0.0.6	https://github.com/sanger-tol/PretextSnapshot
PretextView	1.0.3	https://github.com/sanger-tol/PretextView
purge_dups	1.2.3	https://github.com/dfguan/purge_dups
samtools	1.19.2	https://github.com/samtools/samtools
sanger-tol/ascc	0.1.0	https://github.com/sanger-tol/ascc
sanger-tol/blobtoolkit	0.4.0	https://github.com/sanger-tol/blobtoolkit
sanger-tol/curationpretext	1.4.2	https://github.com/sanger-tol/curationpretext
Seqtk	1.4-r122	https://github.com/lh3/seqtk
Singularity	3.9.0	https://github.com/sylabs/singularity
TreeVal	1.4.0	https://github.com/sanger-tol/treeval
YaHS	1.1a.2	https://github.com/c-zhou/yahs

### Author information

Contributors are listed at the following links:

- Members of the University of Oxford and Wytham Woods Genome Acquisition Lab
- Members of the Natural History Museum Genome Acquisition Lab
- Members of the Marine Biological Association Genome Acquisition Lab
- Members of the Darwin Tree of Life Barcoding collective
- Members of the Wellcome Sanger Institute Tree of Life Management, Samples and Laboratory team
- Members of Wellcome Sanger Institute Scientific Operations – Sequencing Operations
- Members of the Wellcome Sanger Institute Tree of Life Core Informatics team
- Members of the Tree of Life Core Informatics collective
- Members of the Darwin Tree of Life Consortium

### Wellcome Sanger Institute – Legal and Governance

The materials that have contributed to this genome note have been supplied by a Darwin Tree of Life Partner. The submission of materials by a Darwin Tree of Life Partner is subject to the **‘Darwin Tree of Life Project Sampling Code of Practice’**, which can be found in full on the Darwin Tree of Life website. By agreeing with and signing up to the Sampling Code of Practice, the Darwin Tree of Life Partner agrees they will meet the legal and ethical requirements and standards set out within this document in respect of all samples acquired for, and supplied to, the Darwin Tree of Life Project. Further, the Wellcome Sanger Institute employs a process whereby due diligence is carried out proportionate to the nature of the materials themselves, and the circumstances under which they have been/are to be collected and provided for use. The purpose of this is to address and mitigate any potential legal and/or ethical implications of receipt and use of the materials as part of the research project, and to ensure that in doing so we align with best practice wherever possible. The overarching areas of consideration are:

- Ethical review of provenance and sourcing of the material
- Legality of collection, transfer and use (national and international)

Each transfer of samples is further undertaken according to a Research Collaboration Agreement or Material Transfer Agreement entered into by the Darwin Tree of Life Partner, Genome Research Limited (operating as the Wellcome Sanger Institute), and in some circumstances, other Darwin Tree of Life collaborators.
