# Supplementary material for: The genome sequence of the Garden Ectemnius, Ectemnius cavifrons (Thomson, 1870) (Hymenoptera: Crabronidae)
Source: Wellcome Open Res. 2026 Sep 28;11:774. [Version 1] doi: 10.12688/wellcomeopenres.27971.1 (PMC13675622; doi:10.12688/wellcomeopenres.27971.1)
Supplement: Peer review history

# Reviewer report: Reviewer response for version 1

**DOI:** 10.21956/wellcomeopenres.30773.r171311

**Published:** 2026-10-16

**Authors:** Sharu Paul Sharma

**Affiliations:** 1 Iowa State University, Iowa, USA

The article reports a chromosome-level genome assembly for the Garden Ectemnius, Ectemnius cavifrons, produced through the Darwin Tree of Life project. The nuclear genome was assembled from PacBio HiFi reads generated from a male specimen, with Hi-C data from a female specimen used for scaffolding. RNA sequencing supported gene annotation. The assembly is 225.31 Mb, with 93.48% assigned to 14 chromosomal pseudomolecules. The authors also report a 21.69 kb mitochondrial genome and an Ensembl annotation containing 10,644 protein-coding genes and 450 non-coding genes.

 The manuscript provides a useful reference resource for comparative genomics and further investigation of this species.

 Assembly metrics provided in the snailplot, including the BUSCO identification of 95.7% and annotation of the 16,737 transcripts, all point to high quality of the data.

Are the datasets clearly presented in a usable and accessible format, and the assembly and annotation available in an appropriate subject-specific repository?

Yes

Are sufficient details of the sequencing and extraction, software used, and materials provided to allow replication by others?

Yes

Are the rationale for sequencing the genome and the species significance clearly described?

Yes

Are the protocols appropriate and is the work technically sound?

Yes

Reviewer Expertise:

Bioinformatics

I confirm that I have read this submission and believe that I have an appropriate level of expertise to confirm that it is of an acceptable scientific standard.

# Reviewer report: Reviewer response for version 1

**Published:** 2026-09-28

**Authors:** Automated Benchmarking

**Affiliations:** 1 Wellcome Sanger Institute, Hinxton, UK

As this article is a Genome Note, Automated Benchmarking has been used to determine whether the reported assembly passes four defined checks established by the Earth BioGenome Project: the raw and assembled data are publicly available, the Earth BioGenome Project three-part metric is met, the k-mer completeness exceeds 90%, and the BUSCO score passes the stated threshold. For Ectemnius cavifrons (iyEctCavi1), all four checks passed. Data available: pass. ENA study PRJEB66041, raw-read accessions ERR12055576, ERR12071250, and ERR12071251, and assembly accession GCA_964023945.1 are public. Earth BioGenome Project three-part metric: pass. The primary assembly is reported as 6.C.Q63, meeting the 6.C.Q40 reference standard. k-mer completeness: pass. The haploid assembly has 99.43% k-mer completeness, exceeding the Earth BioGenome Project target of 90%. BUSCO: pass. The haploid assembly, which is proposed as the reference assembly for this species, has 95.7% complete BUSCOs (95.7% single-copy and 0.0% duplicated). BUSCO software version: 5.5.0; lineage dataset: hymenoptera_odb10. It meets the target of greater than 90% complete and single-copy BUSCOs. This decision uses values reported in the Genome Note and public accession records.

Reviewer Expertise:

NA

I confirm that I have read this submission and believe that I have an appropriate level of expertise to confirm that it is of an acceptable scientific standard.
